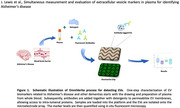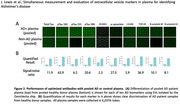# Single step capture and assessment of multiple plasma extracellular vesicle biomarkers in Alzheimer’s disease detection

**DOI:** 10.1002/alz.095648

**Published:** 2025-01-09

**Authors:** Jean M Lewis, Dorathy Ann Harris, Janell Kosmatka, Heath I Balcer, Harmeet Dhani, Juan Pablo Hinestrosa, Paul R Billings, Robert A. Rissman

**Affiliations:** ^1^ Biological Dynamics, San Diego, CA USA; ^2^ University of Southern California, San Diego, CA USA; ^3^ University of California, San Diego, La Jolla, CA USA; ^4^ Alzheimer’s Therapeutic Research Institute, Keck School of Medicine, University of Southern California, San Diego, CA USA

## Abstract

**Background:**

Blood tests for Alzheimer’s disease (AD) that measure biomarkers could be useful as minimally‐invasive ways to give patients more and earlier access to screening. While some AD biomarkers can be detected in plasma, they need to be more sensitive to make plasma AD tests more effective. Extracellular vesicles (EVs) in plasma carry AD‐related biomarkers from the brain and could offer a concentrated source of brain‐related biomarkers, but it is hard to isolate and analyze plasma EVs for clinical use.

**Method:**

We present a simplified method for isolating EVs directly from plasma using an alternating current electrokinetic (ACE) microchip. No sample pre‐treatment steps were needed. Protein biomarkers on the EVs were detected by adding fluorescent antibodies to the plasma samples before capture by the chip. This allowed measurement of EV biomarker levels directly on the chip.

**Result:**

AD or non‐AD control plasma was measured for ten different AD‐related biomarkers. EV‐associated NCAM1, pTau231, α‐synuclein, and TDP‐43 levels were able to distinguish a group of 10 AD, 10 MCI, and 10 non‐AD subjects. pTau231 was different between AD and non‐AD (p = 0.0300) and α‐synuclein differentiated AD from MCI (p = 0.0148).

**Conclusion:**

This study shows how ACE microfluidic chip technology can help differentiate AD and MCI patients from non‐AD controls with clinical relevance. This work also highlights the important diagnostic role of plasma EV biomarkers in neurodegenerative disease.